# ﻿Three novel woody litter inhabiting fungi in Didymosphaeriaceae, Phaeoseptaceae and Synnemasporellaceae from Zhujiangyuan Nature Reserve, Yunnan Province, P.R. China

**DOI:** 10.3897/mycokeys.106.123105

**Published:** 2024-06-21

**Authors:** Gui-Qing Zhang, Nalin N. Wijayawardene, Li-Hong Han, Jaturong Kumla, Nakarin Suwannarach, Qiang Li, Abdallah M. Elgorban, Ihab M. Moussa, Claudia Coleine, Dong-Qin Dai

**Affiliations:** 1 Center for Yunnan Plateau Biological Resources Protection and Utilization, College of Biological Resource and Food Engineering, Qujing Normal University, Qujing, Yunnan Province 655011, China; 2 Tropical Microbiology Research Foundation, 96/N/10, Meemanagoda Road, 10230 Pannipitiya, Sri Lanka; 3 Center of Excellence in Microbial Diversity and Sustainable Utilization, Chiang Mai University, Chiang Mai, Thailand; 4 Center of Excellence in Biotechnology Research, King Saud University, Riyadh, Saudi Arabia; 5 Department of Botany and Microbiology, College of Science, King Saud University, Riyadh 11451, Saudi Arabia; 6 Department of Ecological and Biological Sciences, University of Tuscia, Viterbo, Italy

**Keywords:** Morpho-molecular, new fungal species, phylogeny, taxonomy, woody fungi

## Abstract

Zhujiangyuan Nature Reserve, located in Qujing City, Yunnan Province, China, is reported with high fauna and floral diversity, while the fungal diversity of the region is poorly documented. During the summer season in 2023, decaying wood-inhabiting microfungi were collected from different microhabitats. The novel species were identified based on morphological characteristics and phylogenetic analyses (based on combined datasets of ITS, LSU, SSU, *tef*1-α, and *rpb*2 regions). Two species belong to Dothideomycetes (*viz.*, *Spegazziniazhujiangyuanensis***sp. nov.** and *Phaeoseptumzhujiangyuanense***sp. nov.** in Pleosporales) while the other one resides in Sordariomycetes (*Synnemasporellafanii***sp. nov.** in Diaporthales). The results are in conformity with the earlier studies that predicted higher fungal diversity in this region.

## ﻿Introduction

Fungi have a worldwide distribution and underpin nearly all life on the Earth (Mueller and Schmit 2007). They can grow in a wide range of habitats, including extreme environments like deserts or high salt concentrations (Raghukumar and Raghukumar 1998; Dadachova et al. 2007). Fungi exist in various lifestyles, including pathogenic, saprophytic, endophytic, and symbiotic (Naranjo-Ortiz and Gabaldón 2019). They occur as decomposers to degrade organic materials, contribute to carbon and nutrient cycling directly in ecosystems (Richards et al. 2017), and play a role in facilitating mineral cycling, accelerating rock weathering, and promoting plant growth. Currently, the estimates of fungal diversity range from 2 to 3 million. Species Fungorum (2024) (accession date: 31 May 2024) lists all accepted species of fungi, currently 161,104 species; therefore, over 90% of fungal species is still unknown (Hawksworth and Lücking 2017; Niskanen et al 2023). It is predicted that a number of novel taxa could be harboured in tropical regions where the environmental factors are favourable for higher diversity and continued living (Hawksworth and Lücking 2017). Wijayawardene et al. (2021) reported that Yunnan and Guizhou Provinces in China would be an important locality to explore novel taxa although it showcases subtropical climate.

The Zhujiangyuan Nature Reserve harbours abundant plant resources, with forest coverage of more than 95% and exceeding 1,000 species of plants (Wang et al. 2015). The warm climate and sufficient moisture guarantee a rich fungal diversity in Zhujiangyuan Nature Reserve. However, few studies have been carried out in the Zhujiangyuan Nature Reserve, especially on the floristic diversity of fungi.

Zhujiang is the third longest river in China, which covers about 450,000 km^2^, and flows through most cities in Southern China and a wide range of areas in Northern Vietnam (Guo et al. 2023). It originates from Maxiong Mountain in Zhanyi District, Qujing City, Yunnan Province (Wang 2014). The fungal diversity of this region (i.e. Qujing City and Zhujiangyuan Nature Reserve) is not well documented. Nevertheless, recently, Doilom et al. (2021) introduced *Praeclarispora* Doilom, W. Dong, K.D. Hyde & C.F. Liao, a novel genus, with *Praeclarisporaartemisiae* Doilom, W. Dong, K.D. Hyde & C.F. Liao as the type species. At the same time, Doilom et al. (2021) reported *Plenodomusartemisiae* A. Karun., Phookamsak & K.D. Hyde as a new collection from *Artemisiaargyi* in Qujing City, Yunnan Province. Wijayawardene et al. (2021) introduced two new species of *Phragmocamarosporium* Wijayaw., Yong Wang bis & K.D. Hyde (*viz.*, *P.magnoliae* and *P.qujingensis*) and one new species of *Lonicericola* Phookamsak, Jayasiri & K.D. Hyde (*viz.*, *L.qujingensis*), collected from *Magnoliagrandiflora* from Qujing Normal University garden, Qujing. Furthermore, five new host/geographical records of different taxa on *Magnoliagrandiflora* collected from Qujing City, were also reported by Wijayawardene et al. (2021), *Botryosphaeriadothidea* (Moug.) Ces. & De Not. and *Sheariaformosa* (Ellis & Everh.) Petr. were reported as new geographical records from China; *Diplodiamutila* (Fr.) Fr. and *D.seriata* De Not. were identified as new host records from *M.grandiflora* in China; while *Angustimassarinapopuli* Thambug. & K.D. Hyde was comfirmed as a new host and geographical record by Wijayawardene et al. (2021), which mentioned it is the first report of *A.populi* from China and on *M.grandiflora*.

During the summer of 2023 (July–September), we collected samples of microfungi associated with decaying wood litter in the North-east gate of Zhujiangyuan Nature Reserve. From the collected samples, we introduce three novel species belonging to *Spegazzinia* Sacc. (i.e. *S.zhujiangyuanensis* in Didymosphaeriaceae Munk, Pleosporales, Dothideomycetes O.E. Erikss. & Winka), *Phaeoseptum* Ying Zhang, J. Fourn. & K.D. Hyde (i.e. *P.zhujiangyuanense* in Phaeoseptaceae Boonmee, Thambugala & K.D. Hyde, Pleosporales, Dothideomycetes) and *Synnemasporella* X.L. Fan & J.D.P. Bezerra (i.e. *S.fanii* in Synnemasporellaceae X.L. Fan & J.D.P. Bezerra, Diaporthales Nannf., Sordariomycetes O.E. Erikss. & Winka) based on morpho-molecular analyses. The new taxa are provided with illustrations and morphological descriptions.

## ﻿Materials and methodology

### ﻿Sample collection

With prior permission of the management of Zhujiangyuan Nature Reserve, located in Qujing City, Yunnan Province, China, decaying wood litter samples were collected in the terrestrial habitats. The samples were stored in separate zip-lock plastic bags and transported to the microbiology laboratory of Qujing Normal University. Geographical information and sample information were recorded. Collections were maintained at room temperature (25 °C) and the samples were examined within 3–5 days.

### ﻿Morphology, isolation and preservation

Fruiting bodies were examined using a Leica S8AP0 stereomicroscope with an HDMI 200C camera (Leica Corporation, Germany). Micro-morphological characters were photographed using an Olympus BX53 compound microscope (Olympus Corporation, Japan) with differential interference contrast (Olympus BX53 DIC compound microscope with an Olympus DP74 camera, Japan). Ascomata and conidiomata were sectioned by hand using a razor blade to obtain thin sections (Dai et al. 2022). All microscopic measurements were made using Tarosoft (R) Image FrameWork software (http://www.tarosoft.in.th/), and the measurements were provided as minimum–maximum values and average values. The photographic plates were edited and provided by using Adobe Photoshop CC 2018 (Adobe Systems, USA) software.

Single spore isolation was performed to obtain pure cultures following the methods described in Senanayake et al. (2020). Germinating spores were photographed, transferred to potato dextrose agar (PDA), and then incubated under the dark at 27 °C to obtain a pure culture, which were photographed to record the different characters. After a week, hyphal tips were transferred into PDA plates and grown at 27 °C in the dark.

Dried herbarium specimens and living cultures were preserved at the Mycological Herbarium of Zhongkai University of Agriculture and Engineering (MHZU) and Zhongkai University of Agriculture and Engineering (ZHKUCC), China. Duplicates of holotypes and type cultures were deposited at the Herbarium of Guizhou Medical University, Guiyang, China (GMB) and Guizhou Medical University Culture Collection (GMBCC) in Guiyang, China. Index Fungorum identifiers (2023) were obtained for the newly introduced taxa.

In the text, the following abbreviations are used: n = a number of ascospores/asci/conidiogenous cells/conidiophores/conidia measured from a given number of specimens, *x̄*¯ = arithmetical average of sizes of all ascospores/asci/conidiogenous cells/conidia.

### ﻿DNA extraction, PCR amplification and sequencing

Fresh cultures were grown on PDA in the dark at 27 °C for 15–30 days. The genomic DNA of the fungus was extracted from fresh cultures according to the specifications of the Biospin Fungal Genomic DNA Extraction Kit (bioflux ®). Both forward and reverse primers were used for the amplification of internal transcribed spacers (ITS), large subunit rDNA (LSU), small subunit rDNA (SSU), translation elongation factor 1-α (*tef*1-α) and RNA polymerase II second largest subunit (*rpb*2) regions are listed in Table 1. A final volume of polymerase chain reaction (PCR) was prepared, including 1 μl of DNA template, 1 μl of each forward and reverse primer, 12.5 μl of 2 × taq PCR Master Mix and 9.5 μl of double-distilled water (ddH_2_O) as described by Dai et al. (2022). The PCR thermal cycling procedure for amplifying ITS, LSU, SSU, *tef*1-α and *rpb*2 regions was run under the conditions presented in Table 2. The PCR products were sent to Shanghai Sangon Biological Engineering Technology & Services Co. (Shanghai, People’s Republic of China) for sequencing. All newly generated sequences were deposited in GenBank and accession numbers were obtained.

**Table 1.** Forward and reverse primers information of ITS, LSU, SSU, *tef*1-α and *rpb*2 regions.

Locus	Primers	Reference
ITS	Forward: ITS5 TCCTCCGCTTATTGATATGC	White et al. (1990)
	Reverse: ITS4 GGAAGTAAAAGTCGTAACAAGG	
LSU	Forward: LROR GTACCCGCTGAACTTAAGC	Vilgalys and Hester (1990)
	Reverse: LR5 ATCCTGAGGGAAACTTC	
SSU	Forward: NS1 GTAGTCATATGCTTGTCTC	White et al. (1990)
	Reverse: NS4 CTTCCGTCAATTCCTTTAAG	
*tef*1-α	Forward: EF1-983F	Rehner and Buckley (2005)
	GCYCCYGGHCAYCGTGAYTTYAT	
	Reverse: EF1-2218R	
	ATGACACCRACRGCRACRGTYTG	
*rpb*2	Forward: fRPB2-5f GAYGAYMGWGATCAYTTYGG	Liu et al. (1999)
	Reverse: fRPB2-7cr CCCATRGCTTGTYYRCCCAT	

**Table 2.** The PCR thermal cycling procedure for amplifying ITS, LSU, SSU, *tef*1-α, and *rpb*2 regions.

ITS, LSU, SSU and *tef*1-α	Initial denaturation 95 °C for 5 min. Followed by 35 cycles, denaturation at 95 °C for 30 s, annealing at 55 °C for 50 s, elongation at 72 °C for 90 s. Final extension at 72 °C for 10 min	Dai et al. (2022)
*rpb*2	Initial denaturation 95 °C for 3 min. Follow by 35 cycles, elongation at 94 °C for 1 min, annealing at 52 °C for 50 s, elongation at 72 °C for 1 min. Final extension at 72 °C for 10 min	Ma et al. (2022)

### ﻿Phylogenetic analyses

Based on blast similarity and related publications, closely related sequences were downloaded from GenBank (Table 3). Single gene sequence alignment was performed by mafft v.7.215 (http://mafft.cbrc.jp/alignment/server/index.html) (Katoh and Standley 2013), and final improvements were done using BioEdit v.7.0.5.2 (Hall 2004). Alignment of ITS, LSU, SSU, *tef*1-α and *rpb*2 regions was combined with MEGA6 version 6.0 (Tamura et al. 2013). The alignment of combined datasets in FASTA format was converted to PHYLIP and NEXUS formats by using ALTER (Alignment Transformation Environment online, http://sing.ei.uvigo.es/ALTER/) (Glez-Peña et al. 2010). The online tool Findmodel (http://www.hiv.lanl.gov/content/sequence/findmodel/findmodel.html) was used to determine the best nucleotide substitution model for each partition data (Dai et al. 2022).

**Table 3.** Names, strain numbers, and corresponding GenBank accession numbers of taxa were used in this study.

**Taxon**	**Strain Number**	**GenBank Accession Numbers**			
		**ITS**	**LSU**	**SSU**	***tef*1-α**
**Didymosphaeriaceae**					
*Alloconiothyriumaptrootii*	CBS 980.95^T^	JX496121	JX496234	N/A	N/A
*A.aptrootii*	CBS 981.95	JX496122	JX496235	N/A	N/A
*A.encephalarti*	CPC: 35980	MN562102	MN567610	N/A	N/A
*Austropleosporaarchidendri*	MFLUCC 17-2429	MK347757	MK347974	MK347863	MK360044
*A.archidendri*	MFLU 22-0042	OP058964	OP059055	OP059006	OP135941
*Bambusistromadidymosporum*	MFLU 15-0057^T^	KP761733	KP761730	KP761737	KP761727
*B.didymosporum*	MFLU 15-0058	KP761734	KP761731	KP761738	KP761728
*Bimurianovae-zelandiae*	CBS 107.79^T^	MH861181	AY016356	AY016338	DQ471087
*Chromolaenicolananensis*	MFLUCC 17-1473^T^	MN325015	MN325003	MN325009	MN335648
*C.nanensis*	MFLUCC 17-1477	MN325014	MN325002	MN325008	MN335647
*C.sapindi*	KUMCC 21-0564^T^	OP058967	OP059058	OP059009	OP135943
*Cylindroaseptosporaleucaenae*	MFLUCC 17-2424^T^	NR_163333	NG_066310	MK347856	MK360047
*C.siamensis*	MFLUCC 17-2527^T^	MK347760	MK347976	MK347866	MK360048
*Deniquelatabarringtoniae*	MFLUCC 11-0422^T^	NR_111779	NG_042696	JX254656	N/A
*Dictyoarthriniumvittalii*	NFCCI4249^T^	MF406218	MF182395	MF622059	MF182398
*D.hydei*	SQUCC 13296 ^T^	MW077145	N/A	MW077161	MW075771
*D.musae*	MFLUCC 20-0105^T^	MT482323	MT482320	MT482326	MT495602
*D.musae*	MFLUCC 20-0106	MT482324	MT482321	MT482327	MT495603
*D.sacchari*	MFLUCC 20-0107	MT482325	MT482322	MT482328	N/A
*D.sacchari*	CBS 529.73	N/A	MH872479	N/A	N/A
*D.thailandicum*	KUMCC 21-0664^T^	OP058965	OP059056	OP059007	N/A
*D.thailandicum*	KUMCC 21-0665	OP058966	OP059057	OP059008	OP135942
*Didymocreasadasivanii*	CBS 438.65^T^	MH858658	DQ384103	N/A	N/A
*Didymosphaeriarubi-ulmifolii*	MFLUCC 14-0023^T^	N/A	KJ436586	NG_063557	N/A
*D.rubi-ulmifolii*	MFLUCC 14-0024	N/A	KJ436585	KJ436587	N/A
*Kalmusiaitalica*	MFLUCC 14-0560^T^	KP325440	KP325441	KP325442	N/A
*K.variispora*	CBS 121517^T^	MH863113	MH874668	N/A	N/A
*K.ebuli*	CBS 123120^T^	KF796674	JN644073	JN851818	N/A
*Kalmusibambusatriseptata*	MFLUCC 13-0232^T^	KY682697	KY682695	KY682696	N/A
*Karstenulalancangensis*	KUMCC 21-0670^T^	OP058969	OP059060	OP059011	N/A
*K.lancangensis*	KUMCC 21-0677	OP058970	OP059061	OP059012	N/A
*Laburnicolahawksworthii*	MFLUCC 13-0602^T^	KU743194	KU743195	KU743196	N/A
*L.muriformis*	MFLUCC 14-0921^T^	KU743200	KU743201	KU743202	N/A
*Letendraeacordylinicola*	MFLUCC 11-0150	KM213996	KM213999	KM214002	N/A
*L.cordylinicola*	MFLUCC 11-0148^T^	NR_154118	NG_059530	KM214001	N/A
*Montagnuladonacina*	KUMCC 21-0653	OP058961	OP059052	OP059003	OP135938
*M.thailandica*	MFLUCC 17-1508^T^	MT214352	NG070949	NG070158	MT235774
*Neokalmusiabrevispora*	KT 1466^T^	LC014573	AB524600	AB524459	AB539112
*N.scabrispora*	KT 1023	LC014575	AB524593	AB524452	AB539106
*Neptunomycesaureus*	CMG12^T^	MK912121	N/A	N/A	MK948000
*Paracamarosporiumfagi*	CPC 24890	KR611886	KR611904	N/A	N/A
*P.fagi*	CPC 24892^T^	KR611887	KR611905	N/A	N/A
*P.anthostomoides*	MFLU 16-0172^T^	KU743206	KU743207	KU743208	N/A
*Paraphaeosphaeriarosae*	MFLUCC 17-2547	MG828935	MG829044	MG829150	MG829222
*P.rosae*	MFLUCC 17-2549^T^	MG828937	MG829046	MG829152	MG829223
*Phaeodothiswinteri*	CBS 182.58	N/A	GU301857	GU296183	N/A
*Pseudocamarosporiumpropinquum*	MFLUCC 13-0544	KJ747049	KJ813280	KJ819949	N/A
*P.pteleae*	MFLUCC 17-0724^T^	NR_157536	MG829061	MG829166	MG829233
*Pseudopithomycesentadae*	MFLUCC 17-0917^T^	N/A	NG_066305	MK347835	MK360083
*P.rosae*	MFLUCC 15-0035^T^	MG828953	MG829064	MG829168	N/A
*Septofusisporathailandica*	KUMCC 21-0647^T^	OP058971	OP059062	OP059013	OP135945
*S.thailandica*	KUMCC 21-0652	OP058972	OP059063	OP059014	N/A
*Spegazziniabromeliacearum*	URM 8084^T^	MK804501	MK809513	N/A	N/A
*S.camelliae*	WNA03	MZ538526	MZ538560	N/A	MZ567102
*S.camelliae*	CMU328^T^	MH734522	MH734521	MH734523	MH734524
*S.deightonii*	MFLUCC 20-0002^T^	MN956768	MN956772	MN956770	MN927133
*S.intermedia*	CBS 249.89^T^	MH862171	MH873861	N/A	N/A
*S.jinghaensis*	KUMCC 21-0495^T^	OP058973	OP059064	OP059015	OP135946
*S.jinghaensis*	KUMCC 21-0496	OP058974	OP059065	OP059016	OP135947
*S.lobulata*	CBS 361.58^T^	MH857812	MH869344	N/A	N/A
*S.musae*	MFLUCC 20-0001^T^	MN930512	MN930514	MN930513	MN927132
*S.neosundara*	MFLUCC 15-0456^T^	KX965728	KX954397	KX986341	N/A
*S.radermacherae*	MFLUCC 17-2285^T^	MK347740	MK347957	MK347848	MK360088
*S.tessarthra*	SH 287	JQ673429	AB807584	AB797294	AB808560
***S.zhujiangyuanensis***	**ZHKUCC 23-1020^T^**	**PP060498**	**PP060512**	**PP060504**	**PP035539**
***S.zhujiangyuanensis***	**GMBCC1002**	**PP067151**	**PP067156**	**PP066043**	**PP068812**
*Tremateiaarundicola*	MFLU 16-1275^T^	KX274241	KX274248	KX274254	KX284706
*T.guiyangensis*	GZAAS01^T^	KX274240	KX274247	KX274253	KX284705
*T.murispora*	GZCC 18-2787^T^	NR_165916	MK972751	MK972750	MK986482
*Verrucoconiothyriumnitidae*	CBS 119209	EU552112	EU552112	N/A	N/A
*Xenocamarosporiumacaciae*	CBS 139895^T^	NR_137982	NG_058163	N/A	N/A
*X.acaciae*	MFLUCC 17-2432	MK347766	MK347983	MK347873	MK360093
**Phaeoseptaceae**					
*Alfoldiavorosii*	CBS 145501^T^	JN859336	MK589354	MK589346	MK599320
*Amorocoelophomacassiae*	MFLUCC 17-2283^T^	NR_163330	NG_066307	NG_065775	MK360041
*Angustimassarinaacerina*	MFLUCC 14-0505^T^	NR_138406	KP888637	NG_063573	KR075168
*A.populi*	MFLUCC 13-0034^T^	KP899137	KP888642	NG_061204	KR075164
*A.quercicola*	MFLUCC 14-0506^T^	KP899133	KP888638	NG_063574	KR075169
*Crassiclypeusaquaticus*	CBS 143643^T^	LC312501	LC312530	LC312472	LC312559
*Decaisnellaformosa*	BCC 25616	N/A	GQ925846	GQ925833	GU479851
*D.formosa*	BCC 25617	N/A	GQ925847	GQ925834	GU479850
*Forliomycesuniseptata*	MFLUCC 15-0765^T^	NR_154006	NG_059659	NG_061234	KU727897
*Gloniopsispraelonga*	CBS 112415	N/A	FJ161173	FJ161134	FJ161090
*Guttulisporacrataegi*	MFLUCC 13-0442^T^	KP899134	KP888639	KP899125	KR075161
*Halotthiaposidoniae*	BBH 22481	N/A	GU479786	GU479752	N/A
*Hysteriumangustatum*	MFLUCC 16-0623	N/A	FJ161180	GU397359	FJ161096
*Lignosphaeriafusispora*	MFLUCC 11-0377^T^	NR_164233	KP888646	N/A	N/A
*Mauritianarhizophorae*	BCC 28866	N/A	GU371824	GU371832	GU371817
*Misturatosphaeriaaurantiacinotata*	GKM 1238^T^	N/A	NG_059927	N/A	GU327761
*Phaeoseptumaquaticum*	CBS 123113^T^	KY940803	JN644072	N/A	N/A
*P.carolshearerianum*	NFCCI-4221^T^	MK307810	MK307813	MK307816	MK309874
*P.carolshearerianum*	NFCCI-4384	MK307812	MK307815	MK307818	MK309876
*P.hydei*	MFLUCC 17-0801^T^	MT240622	MT240623	MT240624	MT241506
*P.mali*	MFLUCC 17-2108^T^	MK659580	MK625197	N/A	MK647990
*P.manglicola*	NFCCI-4666^T^	MK307811	MK307814	MK307817	MK309875
*P.terricola*	MFLUCC 10-0102^T^	MH105778	MH105779	MH105780	MH105781
*P.thailandicum*	MFLU 19-2136	OM293749	OR211590	OM293755	OM305059
*P.thailandicum*	HKAS 106993	OM293750	OM293745	OM293756	OM305060
***P.zhujiangyuanense***	**ZHKUCC 23-1022^T^**	**PP060500**	**PP060514**	**PP060506**	**PP035541**
***P.zhujiangyuanense***	**GMBCC1003**	**PP067152**	**PP067157**	**PP066044**	**PP068813**
*Platystomumcrataegi*	MFLUCC 14-0925^T^	KT026117	KT026109	KT026113	KT026121
*Pleopunctumellipsoideum*	MFLUCC 19-0390^T^	MK804512	MK804517	MK804514	MK828510
*P.pseudoellipsoideum*	MFLUCC 19-0391^T^	MK804513	MK804518	N/A	MK828511
*Pseudoaurantiascomakenyense*	GKM 1195^T^	N/A	NG_059928	N/A	GU327767
*P.cornisporum*	CBS 143654^T^	LC312515	LC312544	LC312486	LC312573
*Ramusculicolathailandica*	MFLUCC 13-0284^T^	KP899141	KP888647	KP899131	KR075167
*Sporormurisporaatraphaxidis*	MFLUCC 17-0742^T^	NR_157546	NG_059880	NG_061296	N/A
*Sulcosporiumthailandicum*	MFLUCC 12-0004^T^	MG520958	KT426563	KT426564	N/A
*Teichosporamelanommoides*	CBS 140733^T^	NR_154632	KU601585	N/A	KU601610
*T.pusilla*	CBS 140731^T^	NR_154633	KU601586	N/A	KU601605
*T.rubriostiolata*	CBS 140734^T^	NR_154634	KU601590	N/A	KU601609
*Thyridariamacrostomoides*	GKM 1033	N/A	GU385190	N/A	GU327776
*T.macrostomoides*	GKM 1159	N/A	GU385185	N/A	GU327778
*T.macrostomoides*	GKM 224N	N/A	GU385191	N/A	GU327777
*Vaginatisporaappendiculata*	MFLUCC 16-0314^T^	KU743217	KU743218	KU743219	KU743220
*Westerdykellaornata*	CBS 379.55	AY943045	GU301880	GU296208	GU349021
**Synnemasporellaceae**					
*Apiosporopsiscarpinea*	CBS 771.79	N/A	AF277130	N/A	N/A
*Apiosporopsis* sp.	Masuya 11Af2-1	N/A	AB669034	N/A	N/A
*Apoharknessiainsueta*	CBS 111377^T^	JQ706083	AY720814	N/A	N/A
*A.insueta*	CBS 114575	N/A	AY720813	N/A	N/A
*A.asterospermum*	CBS 112404	N/A	AB553745	N/A	N/A
*A.asterospermum*	KT2138	N/A	AB553744	N/A	N/A
*Auratiopycnidiellatristaniopsidis*	CBS 132180	JQ685516	JQ685522	N/A	N/A
*Cainiellajohansonii*	Kruys 731	N/A	JF701920	N/A	N/A
*Chapeckianigrospora*	AR 3809	JF681957	EU683068	N/A	N/A
*Chiangraiomycesbauhiniae*	MFLUCC 17-1669^T^	MF190118	MF190064	N/A	MF377604
*C.bauhiniae*	MFLUCC 17-1670	MF190119	MF190065	N/A	MF377603
*Chrysocryptacorymbiae*	CBS 132528	JX069867	JX069851	N/A	N/A
*C.koreana*	CBS 143.97	KX833584	AF408378	KX833684	KX833490
*C.straminea*	CBS 149.22	AY339348	AF362569	KX833704	KX833506
*C.wangiensis*	CBS 132530	JX069873	JX069857	KX833705	KX833509
*Coryneumumbonatum*	AR 3541	N/A	EU683072	N/A	N/A
*C.umbonatum*	MFLUCC 15-1110	MF190121	MF190067	N/A	MF377610
*C.umbonatum*	MFLUCC 13-0658^T^	MF190120	MF190066	N/A	MF377609
*Cryphonectriamacrospora*	CBS 109764	EU199182	AF408340	N/A	EU220029
*C.parasitica*	ATCC 38755	AY141856	EU199123	EU222014	DQ862017
*Cryptodiaportheaesculi*	CBS 109765	DQ323530	AF408342	GU354004	EU199138.2
*C.aesculi*	CBS 121905	EU254994	EU255164	DQ313558	EU219269
*C.betulae*	CBS 109763	EU199180	AF408375	EU221884	EU199139
*C.hypodermia*	AR 3552	EU199181	AF408346	N/A	EU199140
*C.suffusa*	CBS 109750	EU199207	AF408376	EU221945	EU199163
*Cytosporaelaeagni*	CFCC 89633	KF765677	KF765693	KU710919	KU710956
*C.leucostoma*	CFCC 50468	KT732949	KT732968	N/A	N/A
*Dendrostomamali*	CFCC 52102^T^	MG682072	MG682012	MG682052	MG682032
*D.osmanthi*	CFCC 52106^T^	MG682073	MG682013	MG682053	MG682033
*D.quercinum*	CFCC 52103^T^	MG682077	MG682017	MG682057	MG682037
*Diaporthedecedens*	CBS 109772	KC343059	AF408348	N/A	N/A
*D.detrusa*	CBS 109770	KC343061	AF408349	KC343787	N/A
*D.eres*	CBS 109767	KC343075	AF408350	KC343801	N/A
*Diaporthellacorylina*	CBS 121124	KC343004	N/A	N/A	N/A
*Diaporthella* sp.	CN 5	KP205483	N/A	N/A	N/A
*Diaporthella* sp.	CN13	KP205484	N/A	N/A	N/A
*Diaporthosporellacercidicola*	CFCC 51994^T^	KY852492	KY852515	N/A	N/A
*D.cercidicola*	CFCC 51995	KY852493	KY852516	N/A	N/A
*Diaporthostomamachili*	CFCC 52100^T^	MG682080	MG682020	MG682060	MG682040
*D.machili*	CFCC 52101	MG682081	MG682021	MG682061	MG682041
*Disculoideseucalypti*	CPC 17650	JQ685517	JQ685523	N/A	N/A
*D.eucalyptorum*	CBS 132184	NR_120090	JQ685524	N/A	N/A
*Ditopelladitopa*	CBS 109748	EU199187	EU199126	N/A	EU199145
*Erythrogloeumhymenaeae*	CPC 18819	JQ685519	JQ685525	N/A	N/A
*G.gnomon*	CBS 199.53	AY818956	AF408361	EU221885	EU219295
*Harknessiaeucalypti*	CBS 342.97	AY720745	AF408363	N/A	N/A
*Hercosporatiliae*	AR 3526	N/A	AF408365	N/A	N/A
*Hyaliappendisporagalii*	MFLUCC 16-1208	MF190149	MF190095	N/A	N/A
*Juglanconisappendiculata*	D96	KY427139	KY427139	KY427208	KY427189
*J.juglandina*	ME23	KY427150	KY427150	KY427219	KY427200
*J.oblonga*	ME14	KY427151	KY427151	KY427220	KY427201
*J.pterocaryae*	ME20	KY427155	KY427155	KY427224	KY427205
*Lamproconiumdesmazieri*	MFLUCC 14-1047	KX430132	KX430133	MF377592	N/A
*L.desmazieri*	MFLUCC 15-0870	KX430134	KX430135	MF377591	MF377605
*Lasmenia* sp.	CBS 124123	GU797406	JF838338	N/A	N/A
*Lasmenia* sp.	CBS 124124	JF838336	JF838341	N/A	N/A
*Luteocirrhusshearii*	CBS 130776	NR_120254	NG_042770	N/A	N/A
*Macrohilumeucalypti*	CPC 19421^T^	KR873244	KR873275	N/A	N/A
*Melanconiellaellisii*	BPI 878343	JQ926271	JQ926271	JQ926406	JQ926339
*M.spodiaea*	MSH	JQ926298	JQ926298	JQ926431	JQ926364
*Melanconisbetulae*	CFCC 50471	KT732952	KT732971	KT733001	KT732986
*M.itoana*	CFCC 50474	KT732955	KT732974	KT733004	KT732987
*M.marginalis*	CBS 109744	EU199197	AF408373	EU221991	EU219301
*M.stilbostoma*	CFCC 50475	KT732956	KT732975	KT733005	KT732988
*Nakataeaoryzae*	CBS 243.76	KM484861	DQ341498	N/A	N/A
*Ophiodiaporthecyatheae*	YMJ1364	JX570889	JX570891	N/A	JX570893
*Pachytrypeprinceps*	Rogers S	N/A	FJ532382	N/A	N/A
*P.rimosa*	FF1066	N/A	FJ532381	N/A	N/A
*Paradiaportheartemisiae*	MFLUCC 14-0850	MF190155	MF190100	N/A	N/A
*P.artemisiae*	MFLUCC 17-1663	MF190156	MF190101	N/A	N/A
*Phaeoappendisporathailandensis*	MFLUCC 13-0161	MF190157	MF190102	N/A	MF377613
*Phaeodiaportheappendiculata*	CBS 123821	KF570156	KF570156	N/A	N/A
*Phragmoportheconformis*	CBS 109783	DQ323527	AF408377	N/A	N/A
*Plagiostomaeuphorbiae*	CBS 340.78	EU199198	AF408382	N/A	DQ368643
*P.salicellum*	CBS 109775	DQ323529	AF408345	EU221916	EU199141
*Prosopidicolamexicana*	CBS 113530	AY720710	N/A	N/A	N/A
*P.mexicana*	CBS 113529^T^	AY720709	KX228354	N/A	N/A
*Pseudomelanconiscaryae*	CFCC 52110^T^	MG682082	MG682022	MG682062	MG682042
*P.caryae*	CFCC 52111	MG682083	MG682023	MG682063	MG682043
*Pseudoplagiostomaeucalypti*	CBS 124807	GU973512	GU973606	N/A	N/A
*P.eucalypti*	CBS 116382	GU973514	GU973608	N/A	N/A
*Pyriculariagrisea*	Ina168	AB026819	AB026819	N/A	N/A
*Rossmaniaukurunduensis*	AR 3484	N/A	EU683075	N/A	N/A
*Silliaferruginea*	CBS 126567	JF681959	EU683076	N/A	N/A
*Stegonsporiumpyriforme*	CBS 124487	KF570160	KF570160	N/A	KF570190
*Stilbosporamacrosperma*	CBS 121883	JX517290	JX517299	N/A	KF570196
*Sydowiellafenestrans*	CBS 125530	JF681956	EU683078	N/A	N/A
*Synnemasporellaaculeans*	CFCC 52094	MG682086	MG682026	MG682066	MG682046
*S.aculeans*	CFCC 52095	MG682087	MG682027	MG682067	MG682047
***S.fanii***	**ZHKUCC 23-1018^T^**	**PP060496**	**PP060510**	**PP035537**	**PP035545**
***S.fanii***	**GMBCC1001**	**PP067150**	**PP067155**	**PP068811**	**PP084097**
*S.toxicodendri*	CFCC 52097^T^	MG682089	MG682029	MG682069	MG682049
*S.toxicodendri*	CFCC 52098	MG682090	MG682030	MG682070	MG682050

Note: “T” denotes ex-type. Newly generated sequences are indicated in bold. “N/A”: no data available in GenBank.

Maximum-likelihood (ML) analysis was carried out via the online portal CIPRES Science Gateway v. 3.3 (Miller et al. 2010), using RAxML-HPC v.8 on XSEDE (8.2.12) tool, with the default settings but adapted: the GAMMA nucleotide substitution model and 1000 rapid bootstrap replicates.

Bayesian analysis was performed by MrBayes v. 3.0b4 (Ronquist and Huelsenbeck 2003), and the model of evolution was estimated with MrModeltest v. 2.2 (Nylander 2004). The posterior probabilities (PP) (Rannala and Yang 1996; Zhaxybayeva and Gogarten 2002) were determined by the following Markov chain Monte Carlo sampling (MCMC) in MrBayes v.3.0b4 (Huelsenbeck and Ronquist 2001). Six simultaneous Markov chains were run for 1,000,000 generations, with trees sampled every 100^th^ generation. The preburn was set to 5 and the run was automatically stopped when the mean standard deviation of the split frequency reached below 0.01 (Maharachchikumbura et al. 2015).

Figtree v. 1.4.0 (http://tree.bio.ed.ac.uksoftware/figtree/) (Rambaut 2006) was used to view tree. Microsoft Office PowerPoint 2016 (Microsoft Inc., Redmond, WA, USA) was used to edit the phylogram, and then convert it to jpg. file by using the Adobe PhotoShop CC 2018 software (Jiang et al. 2021).

## ﻿Results

### ﻿Phylogenetic analyses

**Phylogenetic analyses of *Spegazzinia***

**Figure 1.**
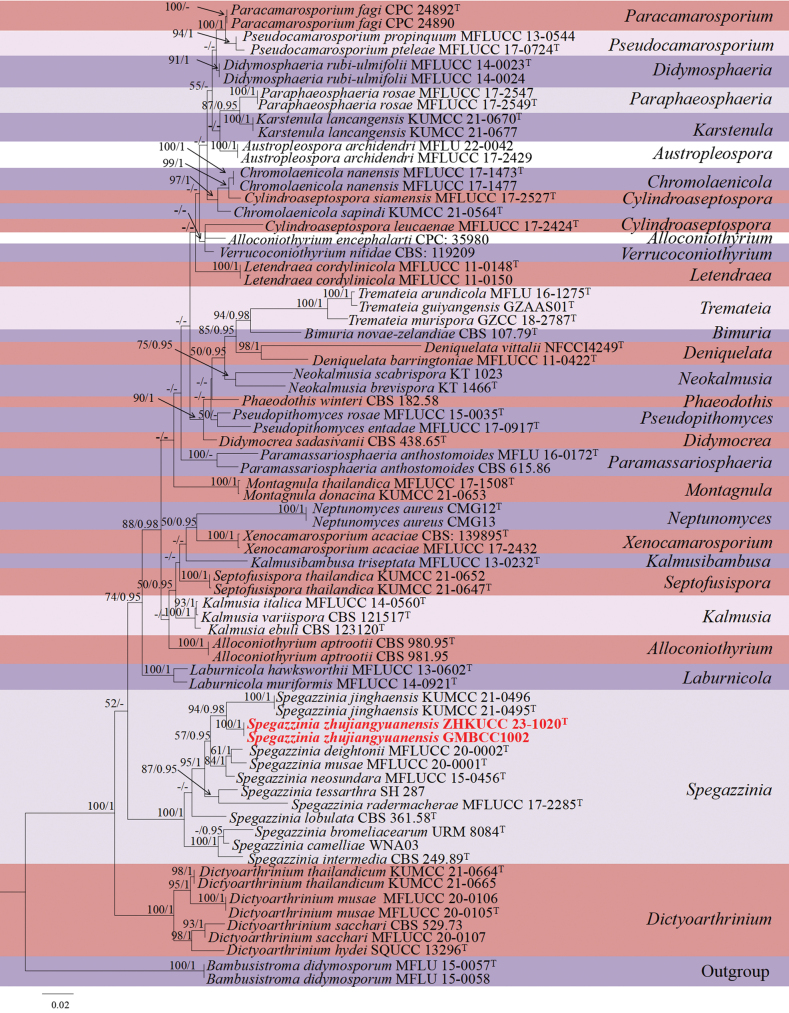
The phylogenetic tree from the best scoring of the RAxML analysis based on combined (ITS, LSU, SSU and *tef*1-α) is rooted to *Bambusistromadidymosporum* (MFLU 15-0057 and MFLU 15-0058). Bootstrap values for maximum likelihood (MLBP) and Bayesian posterior probabilities (BYPP) equal to or greater than 50% and 0.95 are given at the respective branches. Hyphen (-) means a value lower than 50% (BS) or 0.95 (PP). The newly generated sequences are indicated in red bold. The ex-type strains are noted with “T”.

The concatenated dataset (ITS, LSU, SSU, and *tef*1-α regions) contained 74 strains in the sequence analysis, which comprise 2988 characters with gaps. Single gene analysis was carried out and compared with each species, to compare the topology of the tree and clade stability. Two strains of *Bambusistromadidymosporum* D.Q. Dai & K.D. Hyde (MFLU 15-0057 and MFLU 15-0058) are set as the outgroup taxon. The best-scoring RAxML tree with a final likelihood value of -16559.564563 is presented. The matrix had 838 distinct alignment patterns, with 23.64% of undetermined characters or gaps. Estimated base frequencies were as follows; A = 0.238369, C = 0.251538, G = 0.273530, T = 0.236562; substitution rates AC = 1.319072, AG = 2.377467, AT = 1.425866, CG = 0.960524, CT = 6.538802, GT = 1.000000; gamma distribution shape parameter alpha = 0.188509 (Fig. 1). GTR+I+G model was selected as the best model based on MrModeltest and was used for the Bayesian analysis. Overall tree topologies based on ML and BI analyses were similar and not significantly different. In the phylogenetic analysis (Fig. 1), our new strains (ZHKUCC 23-1020 (ex-type) and GMBCC1002) belonged to the genus *Spegazzinia* (Fig. 1). Both strains grouped as the sister clade to *Spegazziniajinghaensis* G.C. Ren & K.D. Hyde (KUMCC 21-0495 (ex-type) and KMUCC 21-0496), and phylogenetically well-distinct with high statistical values (95% ML and 1 PP; Fig. 1).

**Phylogenetic analyses of *Phaeoseptum***

**Figure 2.**
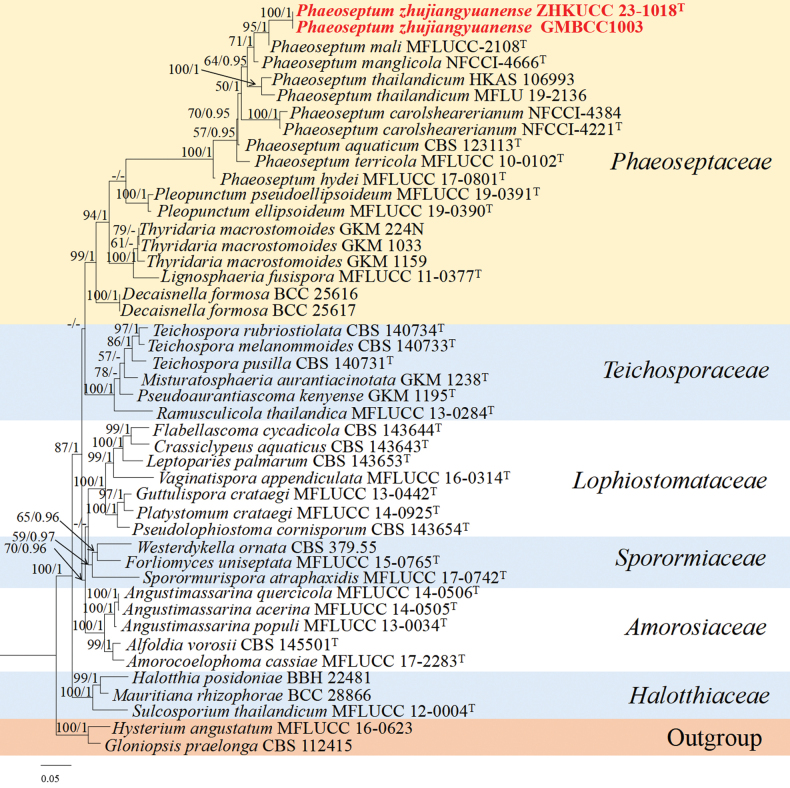
The phylogenetic tree from the best scoring of the RAxML analysis based on combined (ITS, LSU, SSU and *tef*1-α) is rooted to *Hysteriumangustatum* (MFLUCC 16-0623) and *Gloniopsispraelonga* (CBS 112415). Bootstrap values for maximum likelihood (MLBP) and Bayesian posterior probabilities (BYPP) equal to or greater than 50% and 0.95 are given at the respective branches. Hyphen (-) means a value lower than 50% (BS) or 0.95 (PP). The newly generated sequences are indicated in red bold. The ex-type strains are indicated with “T”.

The concatenated dataset (ITS, LSU, SSU, and *tef*1-α regions) contained 45 strains in the sequence analysis, which comprise 3532 characters with gaps. Single gene analysis was carried out and compared with each species, to contrast the topology of the tree and clade stability. *Hysteriumangustatum* Pers. (MFLUCC 16-0623) and *Gloniopsispraelonga* (Schwein.) Underw. & Earle (CBS 112415) were selected as the outgroup taxa. The best-scoring RAxML tree with a final likelihood value of -23164.186742 is presented. The matrix had 1334 distinct alignment patterns, with 25.07% of undetermined characters or gaps. Estimated base frequencies were as follows; A = 0.241078, C = 0.255689, G = 0.276841, T = 0.226392; substitution rates AC = 1.125548, AG = 2.311485, AT = 1.305084, CG = 1.147813, CT = 6.370520, GT = 1.000000; gamma distribution shape parameter alpha = 0.281773 (Fig. 2). GTR+I+G model was selected as the best model based on MrModeltest and was used for the Bayesian analysis. Overall tree topologies based on ML and BI analyses were similar and not significantly different. In the phylogenetic analysis (Fig. 2), two strains of *Phaeoseptumzhujiangyuanense* (ZHKUCC 23-1022 (ex-type) and GMBCC1003) formed a monophyletic clade (100% ML, 1.00 PP). This clade formed a sister taxon to *Phaeoseptummali* Phukhams. & K.D. Hyde (MFLUCC-2108) with 95% ML and 1.00 PP support values.

**Phylogenetic analyses of *Synnemasporella***

**Figure 3.**
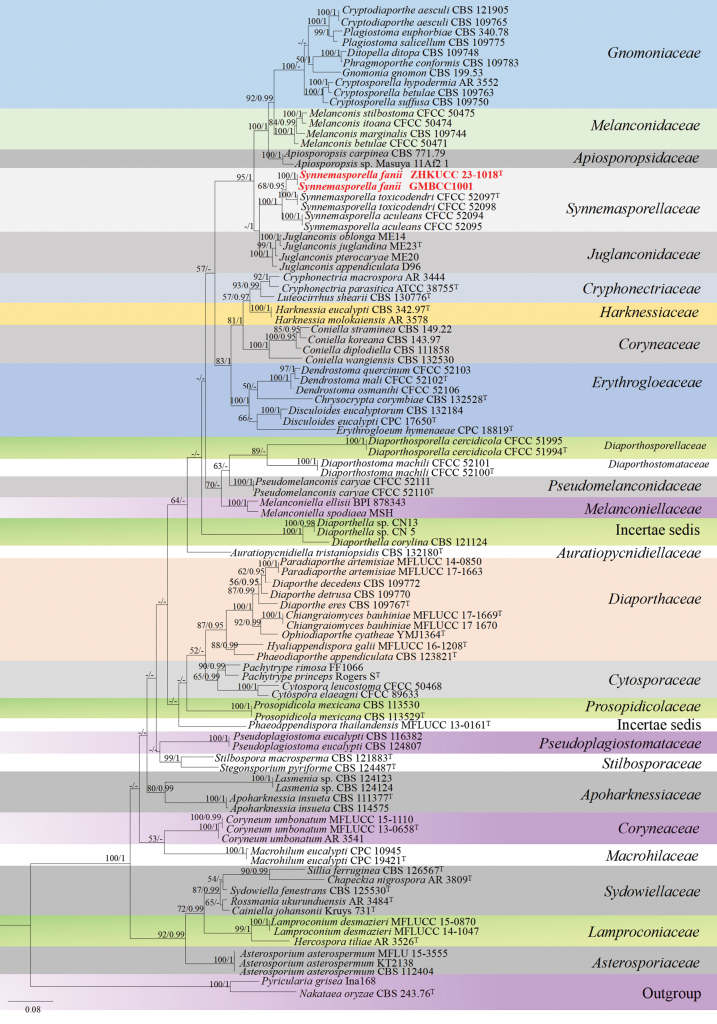
The phylogenetic tree from the best scoring of the RAxML analysis based on combined (ITS, LSU, *tef*1-α and *rpb*2) is rooted to *Nakataeaoryzae* (CBS 243.76) and *Pyriculariagrisea* (Ina168). Bootstrap values for maximum likelihood (MLBP) and Bayesian posterior probabilities (BYPP) equal to or greater than 50% and 0.95, are given at the respective branches. Hyphen (-) means a value lower than 50% (BS) or 0.95 (PP). The newly generated sequences are indicated in red bold. The ex-type strains are indicated with “T”.

The concatenated dataset (ITS, LSU, *tef*1-α and *rpb*2 regions) contained 97 strains in the sequence analysis, which comprise 2575 characters with gaps. Single gene analysis was carried out and compared with each species, to compare the topology of the tree and clade stability. *Nakataeaoryzae* (Catt.) J. Luo & N. Zhang (CBS 243.76) and *Pyriculariagrisea* Cooke ex Sacc. (Ina168) are set as the outgroup taxa. The best-scoring RAxML tree with a final likelihood value of -30093.037277 is presented. The matrix had 1256 distinct alignment patterns, with 32.60% of undetermined characters or gaps. Estimated base frequencies were as follows; A = 0.248601, C = 0.250906, G = 0.280824, T = 0.219669; substitution rates AC = 1.521472, AG = 3.435591, AT = 1.966143, CG = 1.205529, CT = 7.891750, GT = 1.000000; gamma distribution shape parameter alpha = 0.244582 (Fig. 3). GTR+I+G model was selected as the best model based on MrModeltest and was used for the Bayesian analysis. Overall tree topologies based on ML and BI analyses were similar and not significantly different. In the phylogenetic analysis (Fig. 3), our collection of *Synnemasporellafanii* (ZHKUCC 23-1018 (ex-type) and GMBCC1001) resided in the genus *Synnemasporella* and formed a sister clade to *S.toxicodendri* (CFCC 52097 (ex-type) and CFCC 52098) with moderate support (ML 68% and 0.95 PP).

## ﻿Taxonomy

**Class Dothideomycetes O.E. Erikss. & Winka**

**Subclass Dothideomycetidae P.M. Kirk, P.F. Cannon, J.C. David & Stalpers ex C.L. Schoch, Spatafora, Crous & Shoemaker**

**Pleosporales Luttrell ex M.E. Barr**

### ﻿Didymosphaeriaceae Munk

#### ﻿ Spegazzinia

Taxon classificationFungiPleosporalesDidymosphaeriaceae

Sacc.

531AFDB6-2E37-54EE-8588-2A8F48DEF67B

Index Fungorum: IF9963

##### Notes.

The genus *Spegazzinia* was introduced by Saccardo (1880) with *S.ornata* (current name: *S.tessarthra* (Berk. & M.A. Curtis) Sacc. 1886 *fide*Saccardo 1886) as the type species. Initially, based on morphological characters with basauxic conidiogenesis, *Spegazzinia* was accommodated in Apiosporaceae, Sordariomycetes (Hyde et al. 1998). However, Tanaka et al. (2015) transferred *Spegazzinia* to Didymosphaeriaceae (Dothideomycetes) based on molecular data. Morphologically, species of *Spegazzinia* have a distinctive conidiophore ontogeny, as well as two types of conidia: α conidia are composed of 4–8 subglobose, dark cells with long spines, while β conidia are generally subspherical or broadly ellipsoid, flattened in one plane, cruciately septate or muriform, pale brown and smooth-walled (Samarakoon et al. 2020). Currently, 17 epithets are listed in Species Fungorum 2024 (accession date: 31 May 2024). Our new collection morphologically resembles *Spegazzinias. str.* and multi-locus phylogenetic analyses confirmed that it is a novel species.

#### ﻿ Spegazzinia zhujiangyuanensis

Taxon classificationFungiPleosporalesDidymosphaeriaceae

G.Q. Zhang, Wijayaw., & D.Q. Dai sp. nov.

3285B00B-2973-53D1-A45D-453E3AD9D6BA

Index Fungorum: IF901550

Fig. 4

##### Etymology.

Named after the locality from where it was collected, Zhujiangyuan, Yunnan (China).

##### Holotype.

MHCU 23-0273.

##### Description.

***Saprobic*** on twigs of an unknown woody plant. **Sexual morph**: undetermined. **Asexual morph**: Hyphomycetous. ***Conidiomata*** sporodochia, powdery, dark, dense, 0.2–2 mm in diam. ***Conidiogenous cells*** 7–12 µm high × 2.5–6 µm wide (*x̄*¯ = 9.5 × 3.5 µm; n = 10), basauxic, ampulate, subspherical, hyaline-to-light-brown, rough at surface. ***Conidiophores of α conidia*** up to 32.5–142.5 × 1.5–3.5 µm (*x̄*¯ = 82.5 × 2.5 µm, n = 20), erect or flexuous, unbranched, dark brown. ***Conidiophores of β conidia*** 14.5–19 × 2.0–2.3 µm (*x̄*¯ = 16.3 × 2.1 µm; n = 20), short, erect, unbranched, sub-hyaline or light brown. ***Conidia*** two types; ***α conidia*** 17.5–25 × 15.5–26 µm (*x̄*¯ = 20.5 × 19.7 µm; n = 20), 4-celled, stellate-shaped, brown to dark-brown, globose to subglobose, with dark brown warts on the surface of some cells, with conspicuous spines, constricted at septa, 3.6–8 × 1–2.8 µm (*x̄*¯ = 5.3 × 1.7 µm; n = 20); ***β conidia*** 12.2–16 × 12–17 µm (*x̄*¯ = 14.1 × 14.6 µm; n = 20), 4-celled, disc-shaped, quadrangular or subspherical, pale brown at immaturity, becoming brown to dark-brown at maturity, usually attached with conidiogenous cells when detached from the conidiophore, each cell cruciately septate, turbinate, sometimes verrucose around the edges, deeply constricted at septa, flat from side view.

**Figure 4.**
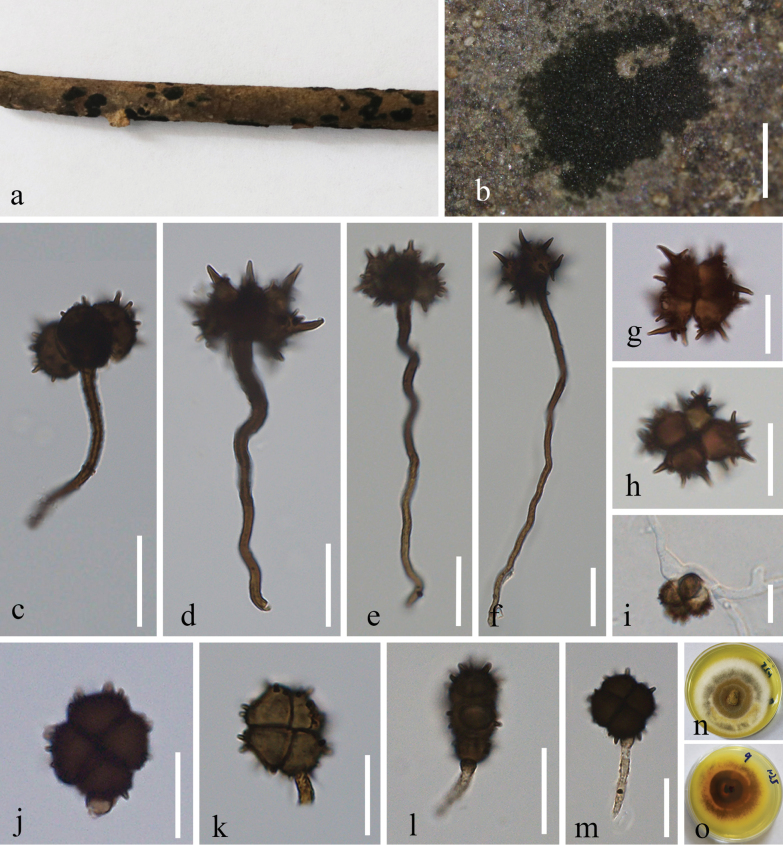
*Spegazziniazhujiangyuanensis* (MHCU 23-0273, holotype) **a, b** fungal colonies on the host surface **c–f** conidiophore of α conidia and α conidia **g, h** α conidia **i** germinated α conidium **j–m** β conidia **n, o** culture characters on pda (**n** above **o** below). Scale bars: 150 μm (**b**); 25 μm (**c**); 20 μm (**d–f**); 15 μm (**g–m**).

##### Culture characteristics.

Conidia germinating on PDA within 24 h. Colonies growing on PDA, reaching reached 30–40 mm diam. After 14 days at 27 °C, superficial, circular, curled, producing concentric circles after 3 weeks, gradually turning brownish gray to white from middle to edge, entire white margin, periphery white at the immature stage, reverse yellowish-brown.

##### Material examined.

China. Yunnan Province, Qujing City, Zhujiangyuan Nature Reserve, 25°30′N, 103°45′E, 01 September 2023, Gui-Qing Zhang & Dong-Qin Dai, QJNU 09 (MHCU 23-0273, ***holotype***), ex-type ZHKUCC 23-1016; *Ibid*. (GMB 1002, **isotype**), ex-isotype GMBCC1002.

##### GenBank numbers.

**Ex-type (ZHKUCC 23-1020)**: PP060498 (ITS); PP060512 (LSU); PP060504 (SSU); PP035539 (*tef*1-α), **ex-isotype (GMBCC1002)**: PP067151 (ITS); PP067156 (LSU); PP066043 (SSU); PP068812 (*tef*1-α).

##### Notes.

Phylogenetic analyses based on ITS, LSU, SSU, and *tef*1-α gene regions showed that our new strains (ZHKUCC 23-1020 (ex-type) and GMBCC1002) belonged to the genus *Spegazzinia* (Fig. 1). Both strains grouped as the sister clade to *S.jinghaensis* (KUMCC 21-0495 (ex-type) and KMUCC 21-0496), but phylogenetically found distinct with high statistical values (95% ML bootstrap and 1.00 PP) (Fig. 1). Morphological differences between the new taxon and *S.jinghaensis* are listed in Table 4. Therefore, based on both morpho-molecular results, we herein introduce a new species in the genus, *Spegazziniazhujiangyuanensis*.

**Table 4.** Diagnostic characters of *Spegazziniajinghaensis* and *S.zhujiangyuanensis*.

Morphological character	Species name and reference	
	*Spegazziniajinghaensis* (Ren et al. 2022)	*S.zhujiangyuanensis* (This study)
Conidiomata	Sporodochial, velvety, 2–3 mm in diam.	Sporodochial, 0.2–2 mm in diam
Conidiogenous cells	5–6 µm long × 4–5 µm wide	7–12 µm long × 2.5–6 µm wide, rough surface
Conidiophores of α conidia	80–120 × 1.4–2 µm, unbranched, dark brown	32.5–142.5 × 1.5–3.5 µm, unbranched, rough surface
Conidiophores of β conidia	3.5–8 × 2.5–3.5 µm short, erect, unbranched, sub-hyaline or light brown	14.5–19 × 2–2.3 µm, short, erect, unbranched, sub-hyaline or light brown
Culture characters	Rough surface, reverse black	With entire white margin, curled, reverse yellowish-brown

### ﻿Phaeoseptaceae S. Boonmee, Thambug. & K.D. Hyde

#### ﻿ Phaeoseptum

Taxon classificationFungiPleosporalesPhaeoseptaceae

Ying Zhang, J. Fourn. & K.D. Hyde

F275368B-9DF8-5CDA-A403-DE699BCA7ABC

Index Fungorum: IF561889

##### Notes.

Zhang et al. (2013) introduced *Phaeoseptum* with *P.aquaticum* Ying Zhang, J. Fourn. & K.D. Hyde as the type species. There are seven *Phaeoseptum* epithets listed in Species Fungorum (31 May 2024). *Phaeoseptum* is characterized by immersed ascomata, cellular pseudoparaphyses, bitunicate, fissitunicate clavate, 8-spored asci, and broadly fusiform, muriform, medium brown coloured, ascospores (Zhang et al. 2013; Phukhamsakda et al. 2019). Our new collection morphologically resembles *Phaeoseptums. str.* The phylogenetic study confirmed that the new collection represents a new species of *Phaeoseptum* (Fig. 2).

#### ﻿ Phaeoseptum zhujiangyuanense

Taxon classificationFungiPleosporalesPhaeoseptaceae

G.Q. Zhang, Wijayaw., & D.Q. Dai sp. nov.

F260ABB0-3241-5D66-A120-C874361B1853

Index Fungorum: IF901551

Fig. 5

##### Etymology.

named after the locality from where it was collected, Zhujiangyuan, Yunnan (China).

##### Holotype.

MHCU 23-0275.

##### Description.

***Saprobic*** on dead wood branches in terrestrial habitats. **Sexual morph: *Ascomata*** 215–470 μm long × 150–320 μm wide (*x̄*¯ = 340 × 225 µm, n = 20), solitary, scattered, semi-immersed to immersed, globose to subglobose, irregular, clypeate, ostiolate, sometimes erumpent as dark brown to black area from the host tissue, or sometimes with a slit-like opening. ***Ostiole*** 33–60 μm high, 15–55 μm diam., short, pale brown. ***Peridium*** 25–60 μm (*x̄*¯ = 44 μm, n = 15) wide, comprising 4–6 layers of cells of *textura angularis*, with thick-walled and brown cells of outer layers, with thin-walled and hyaline cells of inner layers. ***Hamathecium*** composed of 1–1.5 μm (*x̄*¯ = 1.6 µm, n = 20) wide, numerous, branched, cellular, septate, narrow pseudoparaphyses, anastomosing above the asci, and embedded in a gelatinous matrix. ***Asci*** 105–165 × 22–35 μm (*x̄*¯ = 140 × 30 μm, n = 20), 8-spored, bitunicate, fissitunicate, cylindrical-clavate to elongate-clavate, with a distinct pedicel, apically rounded and thinned, with a distinct ocular chamber at immature stage, with a minute ocular chamber when mature. ***Ascospores*** 35–42 × 9–15 μm (*x̄*¯ = 38 × 10 μm, n = 30), partly overlapping, uniseriate at base, 2–3-seriate above, pale to yellowish brown to medium brown from immaturity to maturity, oblong to broadly fusiform, with broadly rounded ends, slightly curved, with 7–13-transversally septa, and 5–21-vertical septa, rarely 2–5 longitudinal septa in each row, normally 1–2 longitudinal septa, but not all cells with a vertical septum in median, the septa partly pale brown, slightly constricted at septa, smooth-walled. Y-shaped septum present or absent in the end cells, with hyaline to pale brown end cells, **Asexual morph**: undetermined.

**Figure 5.**
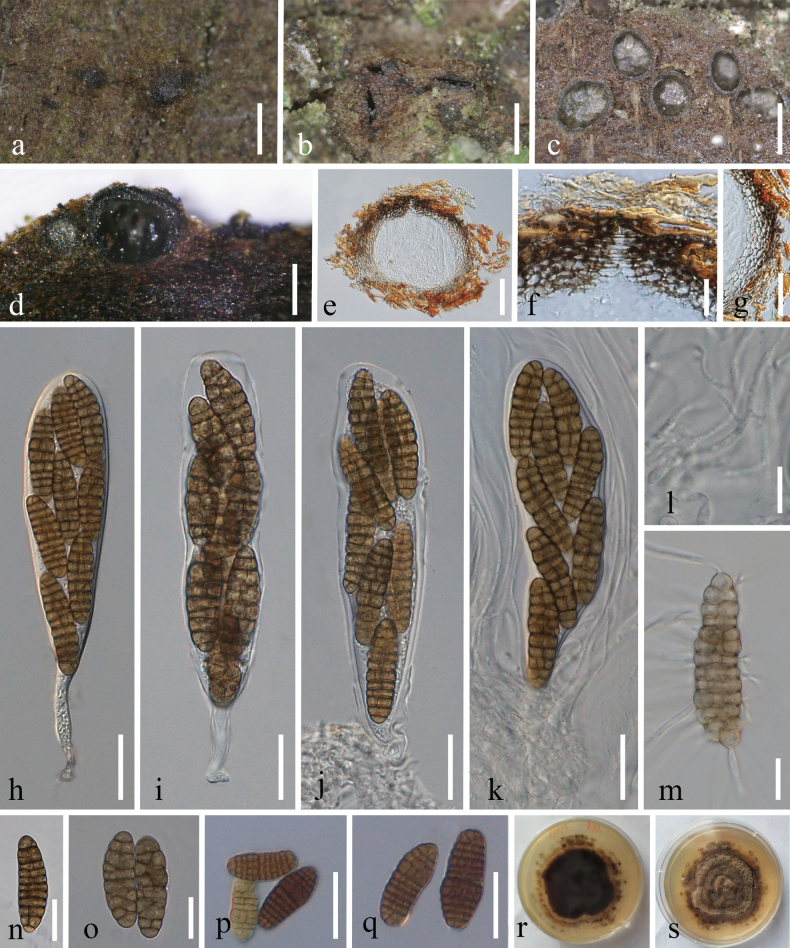
*Phaeoseptumzhujiangyuanense* (MHCU 23-0275, holotype) **a–c** appearance of ascomata on host substrate **d, e** vertical section of ascoma **f** ostiole **g** peridium **h–k** asci **l** pseudoparaphyses **m** germinated ascospore **n–q** ascospores **r, s** colonies on PDA (**r** above **s** below). Scale bars: 300 μm (**a–c**); 200 μm (**d, e**); 50 μm (**f, g, p, q**); 20 μm (**h–o**).

##### Culture characteristics.

Ascospores germinating on PDA, producing germ tubes from both ends of the ascospores within 24 hours. Colonies growing on PDA, reaching reached 30–40 mm diam. after 14 days at 27 °C, surface pale brown, irregular, curled, producing concentric circles after 3 weeks, reverse warm blackish brown with olive buff at margins.

##### Material examined.

China. Yunnan Province, Qujing City, Zhujiangyuan Nature Reserve, 25°30′N, 103°45′E, 01 September 2023, Gui-Qing Zhang & Nalin N. Wijayawardene, RM16 (MHCU 23-0275, ***holotype***), ex-type ZHKUCC 23-1022; *Ibid.* (GMB 1003, ***isotype***), ex-isotype GMBCC1003.

##### GenBank numbers.

**Ex-type (ZHKUCC 23-1022)**: PP060500 (ITS); PP060514 (LSU); PP060506 (SSU); PP035541 (*tef*1-α), **ex-isotype (GMBCC1003)**: PP067152 (ITS); PP067157 (LSU); PP066044 (SSU); PP068813 (*tef*1-α).

##### Note.

The phylogenetic analyses based on a combined dataset of ITS, LSU, SSU and *tef*1-α gene regions (Fig. 2) showed that our isolates (ZHKUCC 23-1022 (ex-type) and GMBCC1003) placed in the genus *Phaeoseptum* in Didymosphaeriaceae (Fig. 2). *Phaeoseptumzhujiangyuanense* clusters with *P.manglicola* (NFCCI-4666) and *P.mali* (MFLUCC-2108) with significant support (ML 100% and 1.00 PP). Morphologically, *P.zhujiangyuanense*, *P.manglicola* Devadatha, V.V. Sarma & E.B.G. Jones and *P.mali* share similar characteristics in their ascomata, asci and ascospores, and in their overlapping dimensions. However, *P.zhujiangyuanense* is distinguishable from *P.mali* and *P.manglicola* in some characters, as shown in Table 5. Therefore, based on both morphological and phylogenetic evidences, we established this novel species in *Phaeoseptum*.

**Table 5.** Diagnostic characters of *Phaeoseptummali*, *P.manglicola* and *P.zhujiangyuanense*.

Morphological character	Species name and reference		
	*P.mali* (Phukhamsakda et al. 2019)	*P.manglicola* (Dayarathne et al. 2020)	*P.zhujiangyuanense* (This study)
Ascomata	Globose ascomata	Globose to subglobose or irregular, aggregate to solitary, with ostiolate	Globose to subglobose, scattered, solitary, ostiolate, with slit-like opening
Ostiole	Opened pore, ostiolate with periphyses	28–94 μm high, 39–96 μm diam	33–60 μm high, 15–55 μm diam
Peridium	5–19 µm, composed of 8–11 layers	30– 85 µm, composed 4–6 layers	25–60 μm wide, composed 4–6 layers
Asci	85–190 × 19–32 μm, cylindrical-clavate to elongate-clavate; apically rounded, ocular chamber clearly visible when immature	102–212 × 17–27.5 μm, cylindrical to clavate; apically rounded and thickened; a refractive plate in the ectoascus and a refractive apical plate in the endoascus	105–165 × 22–35 μm, cylindrical-clavate to elongate-clavate; apically rounded and thinned, with a clearly ocular chamber at immature stage
Ascospores	27–38 × 8–13 μm, broad cylindrical, broadly cylindrical, yellowish to dark brown; 11–14 transverse septa, and 1–2 longitudinal septum in each cell	27–36 × 7.5–13 μm, oblong to broadly fusiform, straight, sometimes slightly curved, hyaline, becoming pale brown to yellowish brown; 9–13 transverse septa, 1–2 longitudinal septa in each row	35–42 × 9–15 μm, oblong to broadly fusiform, slightly curved, pale to yellowish brown to brownness; 7–13-transversally septate, 5–21-vertical septate, 1–5 longitudinal septa in each row

**Sordariomycetes O.E. Erikss. & Winka**

**Diaporthomycetidae Senan., Maharachch. & K.D. Hyde**

**Diaporthales Nannf**

### ﻿Synnemasporellaceae X.L. Fan & J.D.P. Bezerra

Fan et al. (2018) introduced this family to accommodate the holomorphic genus, *Synnemasporella* (with type species *S.toxicodendri* X.L. Fan & J.D.P. Bezerra). Currently, the family comprises only one genus (Wijayawardene et al. 2022a).

#### ﻿ Synnemasporella

Taxon classificationFungiDiaporthalesSynnemasporellaceae

X.L. Fan & J.D.P. Bezerra

244B4403-562E-5341-9B10-9F48C9F1669A

Index Fungorum: IF823995

##### Notes.

The genus *Synnemasporella* is a pleomorphic taxon that exhibits both sexual and asexual morphs (Fan et al. 2018). Currently, the genus comprises two species. The asexual morphs of *S.aculeans* X.L. Fan & J.D.P. Bezerra were reported with both coelomycetous and hyphomycetous morphs (Fan et al. 2018). However, the second species *S.toxicodendri* was reported only with its hyphomycetous morph.

#### ﻿ Synnemasporella fanii

Taxon classificationFungiDiaporthalesSynnemasporellaceae

Wijayaw., G.Q. Zhang & D.Q. Dai sp. nov.

97291E48-5319-5A1A-859F-166DD5549A28

Index Fungorum: IF901552

Fig. 6

##### Etymology.

Named after Dr. Xin-Lei Fan, the mycologist who introduced the genus, to recognize his outstanding contribution to mycology in China.

##### Holotype.

MHCU 23-0271.

##### Description.

***Saprobic*** on twigs of an unknown woody plant. **Sexual morph**: undetermined. **Asexual morph**: hyphomycetous. ***Conidiomata*** synnematous. ***Synnemata*** 1000–1300 µm high, 110–360 µm diam., long and determinate, pale to brown, straight, occasionally curved, composed of parallelly and compactly arranged conidiophores. ***Conidiophores*** 30–70 µm long × 4.5–6.5 µm wide, hyaline to pale brown, aggregated, straight to curved. ***Conidiogenous cells*** 1.5–3.5 × 0.5–2.5 µm, enteroblastic, with a minute collarette at the tip, hyaline to pale brown, straight to curved, cylindrical, arranged adjacent to one another at the fertile end of the synnema, with each conidiogenous cells producing one conidium. ***Conidia*** 23–37 × 11–17 µm (*x̄*¯ = 30 × 15 µm, n = 20), cylindrical to oblong-cylindrical, 1–3 septate, slightly constricted at septa, straight to slightly curved, with a discrete hilum, smooth-walled, multiguttulate, pale brown to brown.

**Figure 6.**
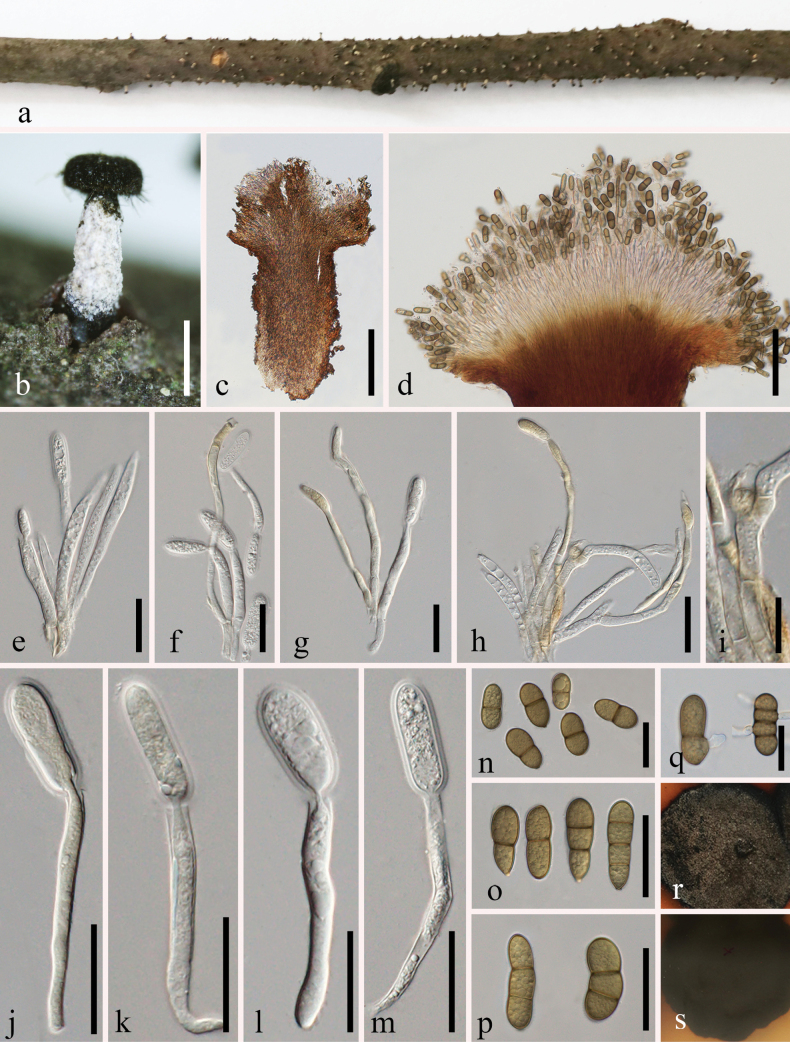
*Synnemasporellafanii* (MHCU 23-0271, holotype) **a, b** habit of synnemata on branches **c, d** longitudinal section of synnemata **e–h** conidiophores and conidiogenous cells **i** conidiophores showing septa **j–m** conidiogenous cells. **n–p** conidia **q** germinating conidia **r, s** colony on PDA (**r** above **s** below). Scale bars: 2 mm (**b**); 300 μm (**c**); 400 μm (**d**); 10 μm (**e, h**); 20 μm (**f, g**); 15 μm (**i**); 25 μm (**j–m, q**); 30 μm (**n–p**).

##### Culture characteristics.

Conidia germinating on PDA within 24 h. Colonies growing on PDA, reaching reached 30–40 mm diam. after 14 days at 27 °C, circular, initially white, becoming sepia on the bottom after 7 days, with an irregular edge, texture uniform.

##### Material examined.

China. Yunnan Province, Qujing City, Zhujiangyuan Nature Reserve, 25°30′N, 103°45′E, 01 September 2023, Gui-Qing Zhang & Nalin N. Wijayawardene, RM17 (MHCU 23-0271, ***holotype***), ex-type ZHKUCC 23-1018; *Ibid.* (GMB 1001, ***isotype***), ex-isotype GMBCC1001.

##### GenBank numbers.

**Еx-type (ZHKUCC 23-1018)**: PP060496 (ITS); PP060510 (LSU); PP035537 (*tef*1-α); PP035545 (*rpb*2), **ex-isotype (GMBCC1001)**: PP067150 (ITS); PP067155 (LSU); PP068811 (*tef*1-α); PP084097 (*rpb*2).

##### Note.

The phylogenetic analyses of the combined dataset of ITS, LSU, *rpb*2 and *tef*1-α gene regions (Fig. 3) showed that our isolates (ZHKUCC 23-1018 (ex-type) and GMBCC1001) belonged to the genus *Synnemasporella* (Fig. 3). *Synnemasporellafanii* clustered with *S.toxicodendri* (CFCC 52097 (isotype) and CFCC 52098) with moderate statistical supports (ML 68% and 0.95 PP). Morphologically, *Synnemasporellafanii* shares similar characteristics in its synnemata with *S.toxicodendri* and *S.aculeans*. Furthermore, *S.fanii* can be distinguished from *S.toxicodendri* and *S.aculeans* by having 1–3-septate conidia. Besides, in both two species of this genus, the form of the conidiogenous cells cannot be discerned well from Fan et al. (2018); it is not certain whether the two species have enteroblastic conidiogenous cells similar to our strain. The other differences are provided in Table 6. Based on morphology and phylogeny, we established this new collection as a novel species of *Synnemasporella*.

**Table 6.** Comparison of morphological characteristics of *Synnemasporella* species.

Morphological character	Species name and reference		
	*Synnemasporellaaculeans* (Fan et al. 2018)	*S.fanii* (This study)	*S.toxicodendri* (Fan et al. 2018)
Synnemata	1100–1500 µm high, 200–400 µm diam., pale to brown, straight to curved, parallel	1000–1300 µm high, 110–360 µm diam., long and determinate, pale to brown, straight, occasionally curved, parallel	1200–1800 µm high, 150–300 µm diam., pale to brown, straight to curved, parallel
Conidiophores	20–30 µm, aggregated, aseptate, straight to curved	30–70 µm long, 4.5–6.5 µm wide, aggregated, septate, straight to curved	20–30 µm, aggregated, aseptate, straight to curved
Conidiogenous cells	Cylindrical, hyaline	Cylindrical, hyaline, enteroblastic, straight to curved	Cylindrical, hyaline
Conidia	8–10(–11) × 3–3.5 µm, oblong-cylindrical, aseptate	23–37 × 11–17 µm, cylindrical to oblong-cylindrical, 1–3 septate, slightly curved	6–8 × 2.5–4 µm, cylindrical to oblong-cylindrical, aseptate
Culture characters	Regular edge; texture initially uniform, producing concentric circle on the margin after 3 days	Irregular edge, circular, initially white, becoming sepia on the bottom after one week	Irregular edge; texture initially uniform, producing concentric circles after 3 weeks

## ﻿Discussion

Zhujiangyuan Nature Reserve in Yunnan Province, China, harbours a large number of native evergreen and deciduous plant species and we predict this region has higher fungal diversity, although many are yet to be discovered (Feng and Yang 2018; Luo et al. 2018; Dai et al. 2019, 2022; Wijayawardene et al. 2021, 2022c). Wijayawardene et al. (2022b) emphasized the importance of collecting materials from under-studied geographical locations as even, some extensively studied hosts could still harbour novel taxa. A few saprobic fungal taxa have been discovered on woody litter in the Zhujiangyuan Nature Reserve but leaf litter inhabiting fungi have been poorly studied in this region. Besides, less attention has been given to saprobic fungi on woody litter in riverine habitats. Thus, a comprehensive study of microfungi in this region is most warranted. Further, morphology-based taxonomic information and phylogenetic sequencing data are needed to clarify their correct taxonomy, phylogeny, and functional biodiversity.

Taxa of Didymosphaeriaceae are often reported as endophytic, pathogenic or saprobic on a wide range of plant hosts (Gonçalves et al. 2019; Hongsanan et al. 2020). Based on the morphology, and phylogenetic analyses, taxa of Didymosphaeriaceae were fairly well-studied and currently, 33 genera have been accepted in Didymosphaeriaceae (Wijayawardene et al. 2022a). However, more new taxa are waiting to be discovered from monotypic genera such as *Barria* Z.Q. Yuan, *Cylindroaseptospora* Jayasiri, E.B.G. Jones & K.D. Hyde, *Kalmusibambusa* Phook., Tennakoon, Thambug. & K.D. Hyde, *Lineostroma* H.J. Swart, *Neptunomyces* M. Gonçalves, T. Vicente & A. Alves, *Vicosamyces* Firmino, A.R. Machado & O.L. Pereira, and *Xenocamarosporium* Crous & M.J. Wingf. In this study, we introduced a novel species of *Spegazzinia*, viz., *S.zhujiangyuanensis* (ZHKUCC 23-1020 (ex-type) and GMBCC1002). Morphologically, our new collections show somewhat similar micro-morphological characters to *S.jinghaensis* (with indistinguishable conidiomata, conidiogenous cells and conidiophores of α conidia), but can be separated by its conidiophores of β conidia). Phylogenetically, our new strains *S.zhujiangyuanensis* (ZHKUCC 23-1020 (ex-type) and GMBCC1002) were grouped as the sister clade to *S.jinghaensis* (KUMCC 21-0495 (ex-type) and KMUCC 21-0496), with distinct, high statistical values (94% ML bootstrap and 1.00 PP) (Fig. 1). Therefore, based on morphological characteristics and phylogenetic evidence (Fig. 1; based on ITS, LSU, SSU, and *tef*1-α regions), we introduce *Spegazziniazhujiangyuanensis* as a new species.

Phaeoseptaceae was introduced by Hyde et al. (2018) to accommodate *Phaeoseptum* (type genus), *Lignosphaeria* Boonmee, Thambug. & K.D. Hyde, and *Neolophiostoma* Boonmee & K.D. Hyde. Currently, Phaeoseptaceae comprises only two genera, i.e. *Phaeoseptum* and *Pleopunctum* N.G. Liu, K.D. Hyde & J.K. Liu (Wijayawardene et al. 2022a). In this study, we introduce a novel species of *Phaeoseptum* (Phaeoseptaceae), *viz.*, *P.zhujiangyuanense*, which shares similar characteristics with *P.mali* and *P.manglicola* in their ascomata, asci and ascospore, and their overlapping dimensions, which fit the characters of *Phaeoseptum* well. However, based on morphological differences (Table 5) and phylogenetic analyses (Fig. 2), our collection can be distinguished from the other known species. Thus, we introduced *P.zhujiangyuanense* as a novel species in *Phaeoseptum*.

Synnemasporellaceae was introduced by Fan et al. (2018) to accommodate the genus *Synnemasporella*. The genus was reported with its both asexual and sexual morphs. The asexual morphs of type species of *Synnemasporella*, *S.toxicodendri* was reported with both coelomycetous and hyphomycetous morphs on the same host material (Fan et al. 2018). However, the second species, *S.aculeans* was reported only with a hyphomycetous morph. However, both species of this genus were not mentioned with the form of conidiogenous cells. In this study, our new species, *S.fanii* is found with only a hyphomycetous morph, which fits well with the characteristics of *Synnemasporella*. *Synnemasporellafanii* shares similarities with *S.toxicodendri* and *S.aculeans* in their synnemata but it can be significantly distinguished by their large-sized, 1–3-septate conidia, and possible enteroblastic conidiogenous cells. *Synnemasporella* is abundant as a hyphomycetous morph but further collections are essential to confirm this assumption.

## Supplementary Material

XML Treatment for Spegazzinia

XML Treatment for Spegazzinia zhujiangyuanensis

XML Treatment for Phaeoseptum

XML Treatment for Phaeoseptum zhujiangyuanense

XML Treatment for Synnemasporella

XML Treatment for Synnemasporella fanii

## ﻿Additional information

### Author ORCIDs

Gui-Qing Zhang https://orcid.org/0000-0001-5354-0607

Nalin N. Wijayawardene https://orcid.org/0000-0003-0522-5498

Li-Hong Han https://orcid.org/0000-0002-6127-0915

Jaturong Kumla https://orcid.org/0000-0002-3673-6541

Nakarin Suwannarach https://orcid.org/0000-0002-2653-1913

Qiang Li https://orcid.org/0000-0002-9735-8214

Abdallah M. Elgorban https://orcid.org/0000-0003-3664-7853

Ihab M. Moussa https://orcid.org/0000-0001-9050-2079

Claudia Coleine https://orcid.org/0000-0002-9289-6179

Dong-Qin Dai https://orcid.org/0000-0001-8935-8807

### Data availability

All of the data that support the findings of this study are available in the main text.
